# Feasibility Analysis of Magnetic Navigation for Vehicles

**DOI:** 10.3390/s19245410

**Published:** 2019-12-09

**Authors:** Dongyan Wei, Lichen Huang, Xinchun Ji, Wen Li, Yi Lu, Hong Yuan

**Affiliations:** 1 Aerospace Information Research Institute, Chinese Academy of Science, Beijing 100864, China; weidy@aircas.ac.cn (D.W.);; 2 School of Electronic, Electrical and Communication Engineering, University of Chinese Academy of Sciences, Beijing 100864, China

**Keywords:** magnetic navigation, feasibility, magnetic maps

## Abstract

Magnetic navigation is a promising positioning technique for scenarios where a global navigation satellite system (GNSS) is unavailable, such as for underwater submarines and aircraft in space. For ground scenarios, it faces more challenges, since the magnetic distribution suffers interference from surrounding objects such as buildings, bridges, and vehicles. It is natural to think how feasible it is to apply magnetic matching positioning to vehicles. In this paper, a theoretic distribution model is proposed to analyze the magnetic field around objects such as buildings, bridges, and vehicles. According to the experiments, it is shown that the proposed model matches the experimental data well. In addition, a comprehensive indicator metric is defined in this paper to describe the feasibility of the magnetic matching method based on the statistical characteristics of magnetic maps. The best length of matching window, anti-noise performance, and pre-comparison of positioning accuracy in different regions can be easily derived using the proposed comprehensive indicator metric. Finally, the metric is verified through a drive test using different building densities.

## 1. Introduction

Currently, the global navigation satellite system (GNSS) is widely used for the navigation of vehicles, since it can provide a high-precision and low-cost position service using a GNSS receiver integrated in a navigator or a smart phone. However, GNSS is only available in scenarios where at least four GNSS satellites are visible, and the performance decreases dramatically in signal-blocked scenarios such as tunnels and underground garages. Thus, numerous positioning technologies emerged such as WiFi-based and Bluetooth-based positioning, dead reckoning (DR), visual positioning, and magnetic-based positioning [1]. Compared comprehensively, for magnetic-based positioning, no hardware deployment is needed in advance, and the sensor has low cost and is easy to implement. Magnetic-based positioning also has the advantages of good concealment and strong stability. Due to the above advantages of magnetic-based positioning, magnetic navigation technology can be applied to the navigation of vehicles. First of all, magnetic fields exist everywhere, even when vehicles pass through remote areas. This ensures that magnetic navigation is available in various regions. Secondly, the magnetic navigation of vehicles is insensitive to weather changes. Lastly, magnetic navigation has good concealment. This is beneficial for the navigation of military vehicles. In summary, the magnetic navigation of vehicles has great prospects for development [2]. 

In magnetic navigation, a magnetic matching algorithm is commonly used because of its simple implementation and high precision. The magnetic matching algorithm includes two phases: the offline phase and online phase. The offline phase is a preparation process before actual positioning. It is aimed at constructing a magnetic map with position information and corresponding magnetic data. There are two ways to construct the magnetic map: using a geomagnetic model and collecting data by sensors [3]. A geomagnetic model is often used in the navigation of large objects like submarines and planes, since it has a large scale [4]. When high positioning accuracy is required, such as in the case of vehicle navigation, we should collect magnetic data and position information to construct the magnetic map. The magnetic map can be constructed by adding supplementary magnetic sensors to a street-view collection system like Google Maps and Baidu Maps [5]. The crowd-source based approach can also be used to create magnetic maps [6,7]. During the online phase, which is the actual positioning phase, only magnetic data are collected. When the magnetic data collected fill a window of a fixed length, the matching process starts. The newly collected magnetic data are matched with a magnetic window of the same length in the magnetic map, in order to obtain the estimated position. There are two representative kinds of matching methods in the online phase: the iterated closest contour point algorithm (ICCP) and magnetic correlation matching algorithm (MAGCOM). The ICCP algorithm is aimed at finding the minimum-distance position by solving the transformation equation of the newly collected magnetic data and the magnetic map [8]. In the traditional ICCP algorithm, a reference track is needed. Based on the track, we can use magnetic information to correct trajectory errors and improve positioning accuracy. However, the large computation and high complexity of the ICCP algorithm may result in limitations when dealing with large amounts of data. The MAGCOM algorithm avoids these limitations, and it has strong applicability. Therefore, it is commonly used in magnetic matching [9]. It searches the magnetic map to find which magnetic window in the magnetic map is the most similar to the online magnetic window. Thus, we can get the current estimated position by the relationship between magnetic data and position information in the magnetic map. In this way, the estimated positions can be obtained using a relatively small computation. The way to evaluate the similarity is to calculate the correlation coefficient between the two windows of magnetic data [10]. There are some correlation functions to calculate correlation coefficient such as the mean absolute difference (MAD), normalized product correlation (NPROD), and Hausdorff distance (HD). Simulation experiments showed that the NPROD method has higher matching accuracy and less computation [11]. Therefore, it is widely used in magnetic matching navigation. 

However, there are some open problems in magnetic navigation for vehicles. Firstly, few studies focused on magnetic navigation for vehicles, since many researches focused on submarines and planes [12,13]. Compared to submarines and planes, the magnetic field measured in vehicles is more susceptible to magnetic objects on the ground. These objects (such as buildings, vehicles, bridges) may have a great impact on the magnetic field. This kind of impact can lead to a change in the feasibility of magnetic navigation. Therefore, we need a specific analysis on the distribution characteristics and influencing factors of magnetic fields. Secondly, there is a problem when choosing the appropriate length of matching window (also called matching length) [14]. When the length selected is too short, mismatch often occurs. This decreases the positioning accuracy. In order to improve the accuracy, most existing algorithms choose the matching length as a large value [15]. This may lead to the exaltation of the matching complexity and a reduction in the matching efficiency. Hence, a method is needed for choosing a suitable matching length. In Reference [16], it was pointed out that the matching length is related closely to the feasibility for the navigation of magnetic maps. When the magnetic map is less feasible for navigation, the matching length should be extended. When the magnetic map is more feasible, we can reduce the matching length properly to improve positioning efficiency. Therefore, we consider adjusting matching length based on the feasibility of magnetic maps. Finally, there are areas that are not suitable for magnetic navigation. In these areas, the positioning accuracy is low even when the matching length is large enough. Thus, in the actual positioning process, we can prejudge the feasibility of magnetic maps and add some supplementary measures in areas with low feasibility. However, there is no complete system for quantitatively assessing the feasibility of magnetic maps. In Reference [17], a multiple-attribute decision-making method was used to evaluate feasibility for magnetic navigation based on multiple magnetic characteristics. However, the indicator obtained from this method can only be used for comparison between two magnetic maps. It cannot provide a reference in adjusting the matching length. In Reference [18], Yapeng proposed a method based on calculating the correlation coefficient of a magnetic sequence and its neighbor in the magnetic map. A smaller correlation coefficient denotes higher feasibility. However, describing the feasibility for magnetic navigation using only the correlation coefficient is not enough. Magnetic data with a small variance may have a high mismatching rate, even when the correlation coefficient is small. Thus, a more comprehensive indicator metric is needed.

In this paper, we propose solutions aiming at these problems. The main contributions of this paper are as follows:

(1) We propose a model to evaluate the magnetic field affected by ground objects in vehicle navigation. The distribution of the magnetic field is analyzed using theoretical modeling and experiments. We also discuss impacts caused by ground factors on the feasibility of magnetic navigation and ways to compensate for the impacts. This can be used to improve positioning accuracy.

(2) We present a comprehensive indicator metric to describe the feasibility for magnetic navigation. The metric is based on five statistical characteristics of the magnetic map. Experiments prove that the metric can give a good description of the feasibility of magnetic navigation. We also develop a method to suggest an appropriate matching length based on the metric. 

The paper is organized as follows: Section 2 presents a model to analyze the impacts caused by surrounding ground objects like buildings and vehicles. Section 3 defines a comprehensive indicator metric to evaluate the feasibility of magnetic navigation. Moreover, a method is proposed to give an appropriate matching length. Section 4 discusses special influencing factors in magnetic navigation and their impact on the feasibility of navigation. Section 5 summarizes the full text.

## 2. Model and Distribution of Magnetic Field around a Typical Target

### 2.1. Composition of the Measured Magnetic Field

The measured magnetic field $H_{m}$ is mainly composed of the main magnetic field of the earth and abnormal magnetic fields generated by magnetic objects [19]. The main magnetic field of the earth $H_{e}$ is distributed uniformly. It can be regarded as a stable value. The abnormal magnetic field $H_{a}$ is composed of a permanent magnetic field, induced magnetic field, and random magnetic field. Thus, the composition of the measured magnetic field is as follows:

(1) $$H_{m}=H_{e}+H_{a}=H_{e}+H_{p}+H_{i}+\varepsilon$$

where $H_{p}$ is the permanent magnetic field, $H_{i}$ is the induced magnetic field, and ε is the random magnetic field.

A permanent magnetic field is mainly caused by hard magnetic materials such as steel materials in buildings or vehicles. Generally, it can be regarded as a constant. In Reference [20], experiments were carried out by establishing a scaled model to evaluate permanent magnetic fields. Conclusions showed that the permanent magnetic field accounts for about three-quarters of the abnormal magnetic field. An induced magnetic field is mainly caused by the magnetization of soft magnetic materials under the influence of the earth’s magnetic field. It is related to the earth’s magnetic field and is also affected by the different poses of the magnetic object. Usually, the induced magnetic field can be expressed as follows:

(2) $$H_{i}=\left[\begin{array}{ccc} D_{11} & D_{12} & D_{13}\\ D_{21} & D_{22} & D_{23}\\ D_{31} & D_{32} & D_{33} \end{array}\right]H_{e}$$

where D is a matrix of magnetic field coefficients. When a ferromagnetic object is fixed, each element of the matrix is a constant. A random magnetic field is usually generated by induced currents. It is composed of high-frequency factors, and it has little effect on the measured magnetic field. Thus, Equation (1) can be expressed as follows:

(3) $$H_{m}=(I+D)H_{e}+H_{p}+\varepsilon$$

### 2.2. Model of A Rectangular Object

From the previous section, we can know that the measured magnetic field is composed of the main magnetic field and anomalous field. Because the anomalous field is closely related to the local geography, it is necessary to analyze the impact of the surrounding environment. In magnetic navigation for vehicles, the typical surrounding factors are buildings and other vehicles. Thus, in the next section, we analyze the anomalous magnetic field distribution around buildings and vehicles using modeling and experimental methods.

**Figure 1:**
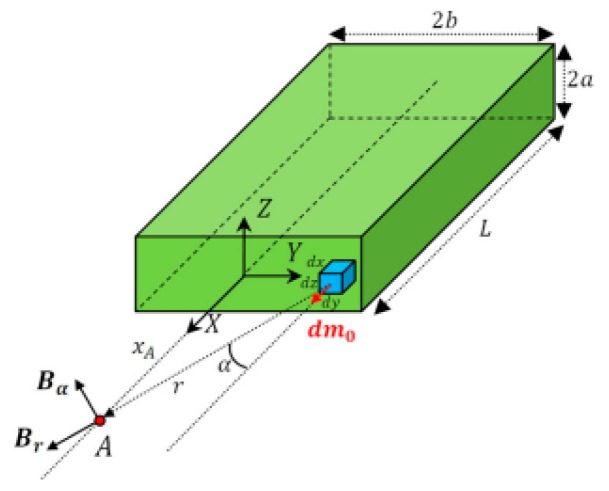
Field generated by a rectangular magnetic object [21].

In Reference [21], a magnetic field model around a rectangular object was established. It was used in analyzing the impact of passing vehicles on the magnetic field. The magnetic field at point A caused by the object is shown in Figure 1. The main function of the model is as follows:

(4) $$B_{x-fs}=\frac{\mu_{0}m_{0}dx}{\pi }\frac{ab(2x_{A}{}^{2}+a^{2}+b^{2})}{(x_{A}{}^{2}+a^{2})(x_{A}{}^{2}+b^{2}){\left(x_{A}{}^{2}+a^{2}+b^{2}\right)}^{1/2}}$$

where $B_{x-fs}$ is the magnetic field at point A generated by the front surface of the object, $\mu_{0}$ is the magnetic constant ($4\pi \times 10^{-7}Vs/Am$), $x_{A}$ is the distance from test point A to the rectangular object in the *x*-direction, $m_{0}$ is the mass of the object, and L, b, and a are the length, width, and height of the object. To obtain the magnetic field caused by the whole rectangular object, $B_{x-fs}$ is integrated as follows:

(5) $$B_{x}=\frac{\mu_{0}m_{0}}{\pi }\int_{-L}^{0}B_{x-fs}$$

### 2.3. Magnetic Field Variations Generated by the Buildings

**Figure 2:**
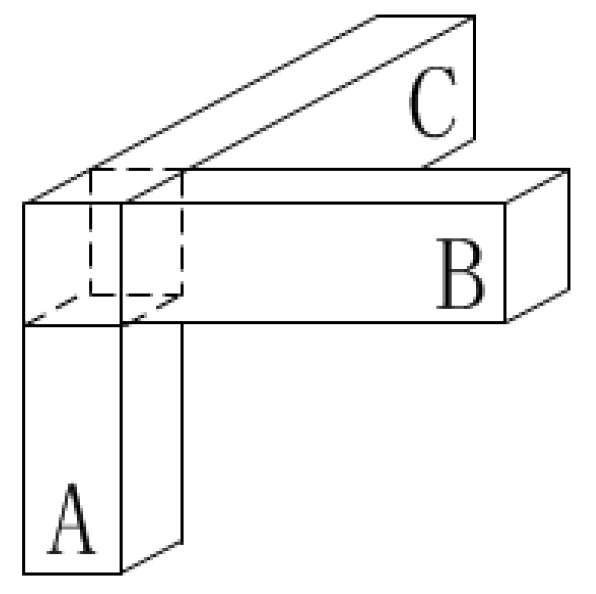
Diagram of simplified steel frame structure.

Since the simple model above was only used in analyzing the magnetic field generated by vehicles [21], it was considered for extension to buildings. The magnetic materials in a building are mainly composed of steel frames and concrete materials. It was pointed out that the magnetic impact generated by steel frames is much greater than that by concrete materials in Reference [22]; thus, the steel frames are mainly discussed. To simplify the steel frame structure, the poses of the steel frame were divided into three types as shown in Figure 2. The magnetic influence produced by a building is a superposition of many steel frames of these three types.

- For steel frames of type A, we can assume a>>b≈L; thus, the expression is as follows:
  (6) $$B_{x-fs}\approx \frac{\mu_{0}m_{0}dx}{\pi }\frac{a^{1/2}b}{\left(x_{A}{}^{2}+b^{2}\right)}$$
  By integrating and simplifying the expression, we can get the following:
  (7) $$B_{xA}=\frac{a^{1/2}\mu_{0}m_{0}}{\pi }\arctan\frac{bL}{b^{2}-(x_{A}+L)x_{A}}\approx p_{1}\arctan\frac{q_{1}}{-x_{A}{}^{2}+Bx_{A}+C_{1}}$$
  where both $p_{1}$ and $q_{1}$ are constants. B and C are parameters related to the size of the frame.
- For steel frames of type B, we can assume b>>a≈L. Since *a* and *b* are symmetrical, the expression is as follows:
  (8) $$B_{xB}=\frac{b^{1/2}\mu_{0}m_{0}}{\pi }\arctan\frac{aL}{a^{2}-(x_{A}+L)x_{A}}\approx p_{2}\arctan\frac{q_{2}}{-x_{A}{}^{2}+Bx_{A}+C_{2}}$$
- For steel frames of type C, we can assume L>>a≈b. The expression is as follows:
  (9) $$B_{xC}=\frac{2\mu_{0}m_{0}}{\pi }[\arctan\frac{L}{{\left(2b^{2}+L^{2}\right)}^{1/2}}-\arctan\frac{x_{A}}{{\left(2b^{2}+x_{A}{}^{2}\right)}^{1/2}}]$$

**Figure 3:**
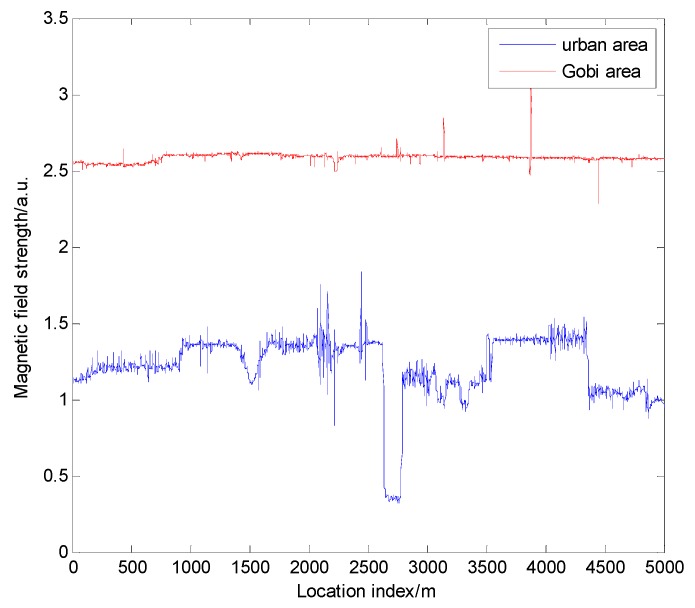
Magnetic field strength in urban area and Gobi area.

Considering $x_{A}+L>>x_{A}$ and $x_{A}<<b$, we can get $B_{xC}\approx 0$. The influence generated by the C-type steel frame is negligible. Thus, the magnetic field strength produced by the steel frame is proportional to $-\tan^{-1}\frac{p}{\left(x^{2}+ax+b\right)}$. This corresponds to the relationship between magnetic field strength and distance proposed in Reference [23]. It means that the above model can not only be used in analyzing vehicles, but also in analyzing buildings. This model can also help analyze the relationship between magnetic field and distance. In Reference [23], it was indicated that the influence range is over 100 m, and the strength is over 10 μT. Therefore, the buildings have a significant, continuous, and stable impact on magnetism. This impact can be regarded as a characteristic factor, which may be beneficial to magnetic matching navigation. In order to verify the above conclusions, the magnetic data were measured separately in an urban area with a dense group of buildings and in the Gobi area with a sparse group of buildings. The equipment used in the experiment was an SBGIG-500N magnetic sensor. The sensor can measure the strength of a three-axis magnetic field, and its sampling frequency can reach 1000 Hz. In order to visually observe the relationship between magnetic strength and location index, we additionally added an odometer to obtain magnetic field and mileage information simultaneously. The magnetic map obtained is shown in Figure 3. The horizontal axis shows the location index of the vehicle, while the vertical axis shows the combined magnetic field strength.

It can be seen from Figure 3 that the magnetic field characteristics in the urban area were more than in the Gobi area. The reason for this phenomenon is the magnetic anomalies generated by urban buildings. The area with more significant magnetic characteristics has greater spatial recognition and, thus, is more suitable for positioning. It was inferred that, in environments where buildings are densely distributed, like in the city, the magnetic map has better feasibility for magnetic navigation. This conclusion can be used for roughly evaluating the feasibility for navigation of a magnetic map.

### 2.4. Magnetic Field Variations Generated by Vehicles

In addition to the effects caused by buildings, magnetic characteristics may be affected by other vehicles. Here, we discuss the magnetic impact caused by a vehicle. The rectangular model was simplified by assuming *b* >> *a* and $x_{A}+L>>x_{A}$ for the vehicle [21]. After simplification, the following equation can be obtained:

(10) $$B_{x}=\frac{\mu_{0}m_{0}a}{\pi x_{A}}$$

Considering the influence of the earth’s main magnetic field, the measured magnetic field at the test point is as follows:

(11) $$B_{x}=\frac{\mu_{0}m_{0}a}{\pi x_{A}}+B_{e}\approx \frac{p}{x_{A}}+q$$

Both *p* and *q* are related to the size and material of the vehicle. It can be seen that the influence caused by the vehicle is inversely proportional to the distance between the test point and the vehicle. 

**Figure 4:**
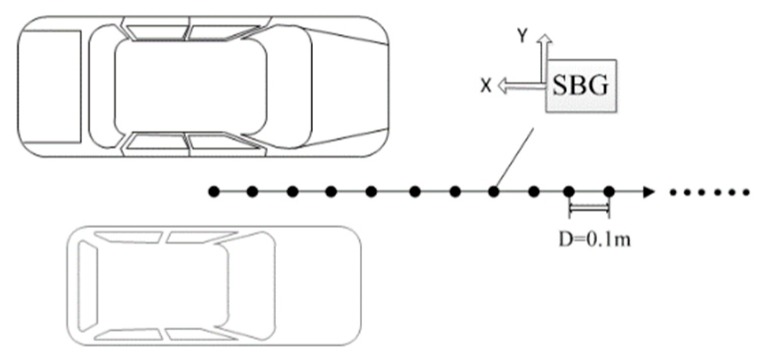
Scenario for measuring the magnetic field caused by a vehicle.

An experiment used to verify the distance model of vehicles was designed. We chose Zhongguancun Forest Park as the test area, because there are no large buildings around. This ensured that the measured magnetic values of different positions were almost the same. Several test points were established along a straight line in the test area. The two test vehicles were parked parallel to a straight line at the starting position of the test points, as shown in Figure 4. Test situations were divided into four cases: car A, car B, car A + car B, and no car at the starting position. The magnetic values of each test point were measured separately to investigate the magnetic changes in these four cases. The vehicles used in the experiment were a Buick sedan and a Benz test car, and the experimental device was an SBGIG-500N magnetic sensor. 

**Figure 5:**
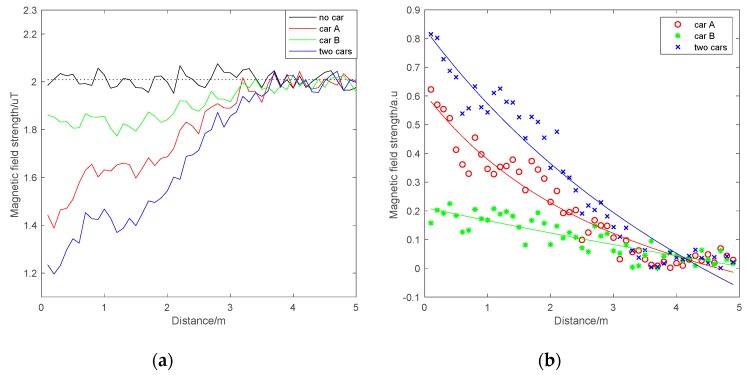
Magnetic field strength in different vehicle environments: (**a**) original measured magnetic field strength; (**b**) magnetic effects caused by vehicle.

The experimental results are shown in Figure 5. The horizontal axis shows the position information, and the vertical axis shows the normalized magnetic field strength. It can be seen that the magnetic values were almost stable (black line) when there was no vehicle in Figure 5a. After parking a vehicle at the starting position, the magnetic field strength had an obvious increase. With the increase in distance, the magnetic field strength quickly returned to the previous stable value. Considering the measurement error learned from the sensor manual, the effective range of the vehicle was about 3 m. Figure 5b shows the magnetic effects caused by the vehicle. It can be seen that the influence generated by the vehicle was approximately inversely proportional to the distance between the test point and the vehicle. The attenuation of this influence was also obvious. The magnetic effect produced by the two cars was approximately the sum of the effects caused by each of the two cars. In actual vehicle magnetic navigation, since magnetic fields caused by other vehicles are almost completely attenuated at about 3 m, the interference from other vehicles is negligible if the vehicle spacing is at a safe distance. If a special situation when overtaking other vehicles occurs, considering the speed of the test vehicle, the time of influence caused by other vehicles is short. A sudden peak may be presented, which can be compensated for by filtering. As for the interference from the carrier itself, there are some useful solutions to compensate for this effect, such as the modeling method in Reference [24]. Thus, in vehicle navigation, there is no need to take into consideration the influence generated by other vehicles. This can increase the stability of magnetic navigation.

## 3. Feasibility for Magnetic Navigation of Magnetic Maps

This section aims to present an indicator metric to evaluate the feasibility for the magnetic navigation of magnetic maps. Since the feasibility is related to the statistical characteristics of a magnetic map, and different characteristics have different characterizing capabilities, we consider presenting an indicator metric based on various statistical characteristics. There are some studies that analyzed the capabilities of these characteristics. In Reference [25], the importance of magnetic characteristics was analyzed by genetic algorithm. It was pointed out that the temporal shape characteristics are the most appropriate to characterize the behavior of the magnetic field signal. In Reference [26], the importance of magnetic characteristics was analyzed by the characteristic removal method. The conclusion showed that the standard deviation, fractal dimension, and magnetic entropy were the three important parameter indicators. Reference [27] analyzed 13 statistical characteristics of magnetic maps, and the authors proposed that Fisher information is also an important factor which can affect the feasibility for magnetic navigation. However, the above analysis did not consider the influence of the anti-noise ability of the magnetic map. Reference [18] pointed out that the anti-noise ability of a magnetic map has a great impact on its feasibility for magnetic navigation. Therefore, based on the above considerations, we chose standard deviation, Fisher information, magnetic entropy, correlation coefficient, and anti-noise ability as an indicator set, and then used the analytic hierarchy process (AHP) to obtain a weighted final comprehensive indicator metric to evaluate the matching feasibility.

### 3.1. Magnetic Map Characteristics

In this section, we introduce the magnetic map characteristics in the indicator set. Suppose that the magnetic map has one dimension (along the direction of the lane). Then, f(i) is the magnetic value of point *i*, and *n* is the number of measurement points in the map. The characteristics are defined below.

#### 3.1.1. Magnetic Standard Deviation

The magnetic standard deviation reflects the dispersion of a magnetic field in the area. The bigger the standard deviation is, the more obvious the magnetic fluctuation is. Additionally, it is more suitable for positioning. The one-dimensional magnetic standard deviation is calculated as follows:

(12) $$\sigma =\sqrt{\frac{1}{n}\sum_{i=1}^{n}(f(i)-\bar{f}{)}^{2}}$$

where $\bar{f}$ is the mean of f(i) in the magnetic map.

#### 3.1.2. Fisher Information

Fisher information indicates the characteristics of the information contained in the magnetic map. When the Fisher information gets larger, there are more characteristics in the magnetic map. Moreover, it is more suitable for positioning. The calculation of the one-dimensional magnetic Fisher information is shown as follows:

(13) $$FIC=\sqrt{\frac{1}{n}\sum_{i=1}^{n}{\left\|\nabla h(i)\right\|}^{2}}$$

where ∇h(i) is the gradient of point *i*.

#### 3.1.3. Magnetic Entropy

The magnetic entropy reflects the disorder of the magnetic field in this area. The smaller the magnetic entropy is, the more favorable it is to locate. The one-dimensional magnetic entropy is calculated as follows:

(14) $$M=-\sum_{i=1}^{n}p(i)\log_{2}(p(i))$$

where $p(i)=f(i)/(\sum_{i=1}^{n}f(i))$. Since the magnetic entropy is inversely proportional to the positioning performance, 1/M is used as an evaluation index.

#### 3.1.4. Correlation Coefficient

The magnetic correlation coefficient reflects the discrimination between the magnetic fields in different positions. When the correlation coefficient is bigger, it is less suitable for positioning. The calculation of the one-dimensional correlation coefficient using the NPROD algorithm is shown as follows:

(15) $$G=\frac{\sum_{i=1}^{n-1}\left|f(i)-\bar{f}\right|\left|f(i+1)-\bar{f}\right|}{(n-1)\sigma^{2}}$$

where σ is the magnetic standard deviation of the map. Since the correlation coefficient is inversely proportional to the positioning performance, 1/G is used as an evaluation index. 

#### 3.1.5. Anti-Noise Ability

Anti-noise ability is also an important indicator to evaluate whether the magnetic map is suitable for matching. The a of a magnetic map can be obtained by calculating the correlation coefficient of the magnetic map with Gaussian noise, whose variance is the sensor’s measurement error, and the original magnetic map. If the correlation coefficient is large, which means the influence of noise is not significant, the magnetic map has strong anti-noise ability. It is, thus, more suitable for positioning. To eliminate the randomness of noise, we added noise to the magnetic map multiple times and took the mean. The method used to calculate the correlation coefficient of the two magnetic maps was the NPROD algorithm. The correlation coefficient of the one-dimensional magnetic map is calculated as follows:

(16) $$R(f,g)=\frac{\sum_{i=1}^{n}(f(i)-\bar{f}){\left(g(i)-\bar{g}\right)}^{T}}{\sqrt{\sum_{i=1}^{n}{\left(f(i)-\bar{f}\right)}^{2}\sum_{i=1}^{n}{\left(g(i)-\bar{g}\right)}^{2}}}$$

where g(i) is the magnetic value with the Gaussian noise, and f(i) is the original magnetic value.

### 3.2. Matching Probability

The matching probability is defined as the ratio of the number of successful positioning times (the positioning error is within a threshold value) to the total number of positioning times. The threshold value is defined according to actual demand. Here, we used the matching probability as a criterion of the actual evaluation of positioning performance. The greater the matching probability is, the better the positioning performance in the actual positioning process is.

### 3.3. AHP Used to Weight

The analytic hierarchy process (AHP) method is a multi-criteria decision-making method combining qualitative and quantitative propositions proposed by Saaty. Its main idea is to get the weight of every factor based on the importance of the factor [28]. Its core steps are as follows: analyzing the relative importance of each factor; constructing the importance matrix of the factors; obtaining the weight of each factor by calculating the matrix eigenvalues; testing the availability of weights with a consistency check. The construction criteria of the importance matrix are given below. The two indicators are compared in pairs to obtain the relative importance. The relative importance between the indicators is usually expressed by integers 1–9 or their reciprocal, with a total of nine scales. There is also an equation of $a_{ij}=\frac{1}{a_{ji}}$. The method for determining the matrix elements $a_{ij}$ is shown in Table 1.

**Figure 6:**
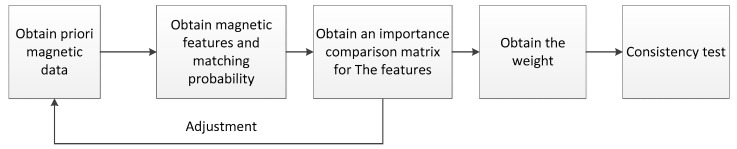
Schematic drawing of AHP process.

In this paper, the AHP method was mainly used to weight the various characteristics in Section 3.1. Its main step was constructing the importance comparison matrix of each indicator and weighting each indicator according to the relative importance of the indicators. Through some a priori magnetic map data, the influence of each characteristic on the final matching probability could be judged, and then the importance comparison matrix was constructed. The AHP process is shown in the Figure 6. 

By comparing the appearing frequencies of the first four features after 200 generations in Reference [25], we could get the relative importance of the first four characteristics. Some a priori positioning simulation experiments with different anti-noise ability were carried out to compare the relative importance degree of anti-noise ability and other characteristics. After finding the correspondence relationship from Table 1, the importance comparison matrix was generated as follows:

(17) $$A=\left[\begin{array}{ccccc} 1 & 4 & 5 & 3 & 2\\ 1/4 & 1 & 2 & 1/2 & 1/4\\ 1/5 & 1/2 & 1 & 1/3 & 1/5\\ 1/3 & 2 & 3 & 1 & 1/3\\ 1/2 & 4 & 5 & 3 & 1 \end{array}\right]$$

Then, the maximum eigenvalue of matrix A and the corresponding eigenvector could be obtained. Then, the weight vector could be obtained by normalizing the eigenvector. The applicable weights were as follows:

(18) W=[0.4065,0.0880,0.0568,0.1418,0.3069]

(19) $$\lambda_{\max}=5.1207$$

The inconsistent comparison matrix is shown below.

(20) $$CR=\frac{\left(\frac{\lambda_{\max}-n}{n-1}\right)}{RI}=\frac{\left(\frac{5.1207-5}{4}\right)}{1.12}=0.0269<0.1$$

From the equation above, we can see the CR satisfies the consistency condition; thus, the weight W is reliable. Therefore, the final magnetic comprehensive indicator metric I obtained is shown in Equation (21).

(21) I=W∗F

where *F* is the set of indicators (standard deviation, Fisher information, magnetic entropy, correlation coefficient, and anti-noise ability).

### 3.4. Experimental Results

**Figure 7:**
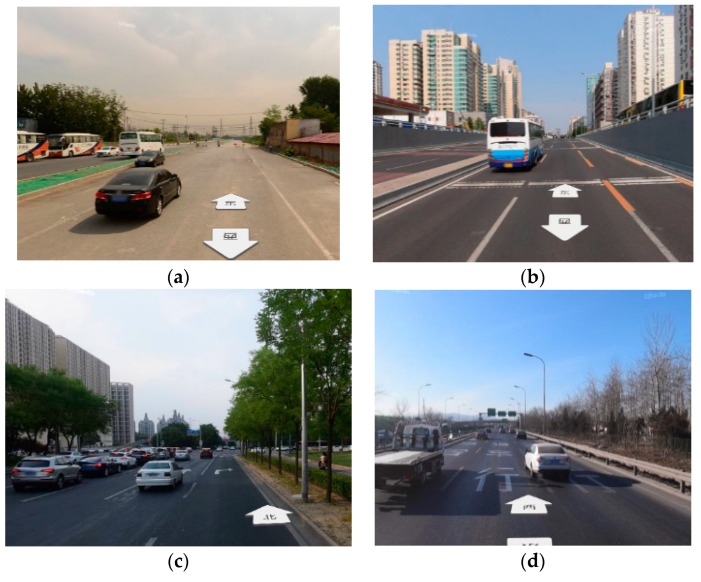
Different experimental scenarios for magnetic matching navigation: (**a**) Scenario 1, country road; (**b**) Scenario 2, city bustling road; (**c**) Scenario 3, city road around a park; (**d**) Scenario 4, highway road.

To discuss the feasibility of the magnetic comprehensive indicator metric I, an experiment was designed. We selected the four scenarios as the test area, as shown in Figure 7. From the figure, we can see that the distribution intensity of the surrounding buildings was in the following order: scenario 2 > scenario 3 > scenario 4 ≈ scenario 1; thus, we could preliminarily guess the feasibility for magnetic navigation of these four scenarios to be in the same order. The selected roads in these scenarios were 500 m long, in order for the roads to have relatively consistent magnetic characteristics. Gaussian white noise was added to the four magnetic maps variances of 1 μT, 3 μT, 5 μT, and 10 μT to constitute the test data, in order to match the test data with the magnetic map. In the four scenarios, the magnetic matching positioning process of the same matching length was performed, and the matching probability was obtained. As shown in the Table 2, it can be seen that, as I increased, the matching probability also increased, and the magnetic map with larger I had higher stability and robustness.

**Figure 8:**
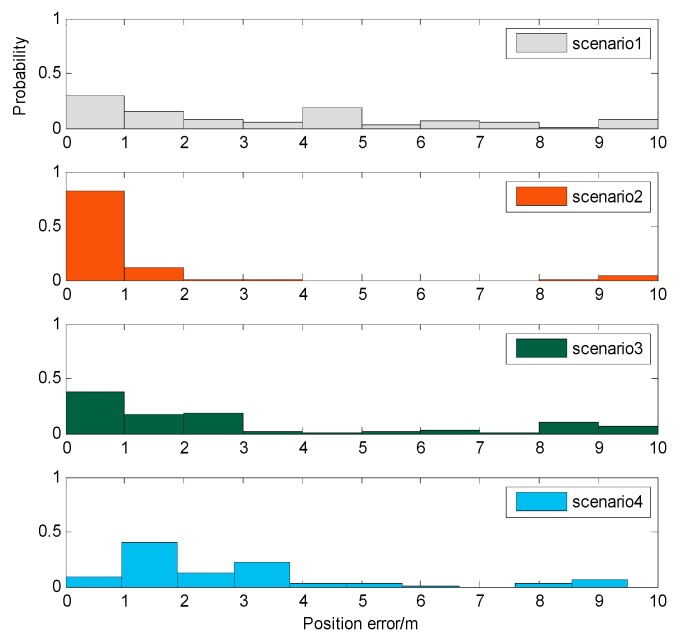
Magnetic positioning results of different road scenarios.

In order to increase the credibility of the magnetic comprehensive indicator metric I, an actual experiment was carried out. Test data were actually collected on the four roads above, and the test data were matched with the magnetic map to obtain the positioning result. To improve the searching efficiency, we added some constraints to the magnetic map searching range. The current position was limited to 10 m from the previous positioning result. The experimental results obtained by the positioning test are shown in Figure 8. 

The *x*-axis shows the positioning error, and the *y*-axis shows the probability of the positioning error being in a certain range. It can be seen that, in the actual test, the location performance of the road segment was in the following order: scenario 2 > scenario 3 > scenario 4 > scenario 1. This result is consistent with the conclusion obtained by the magnetic characteristic indicator I. Therefore, it can be judged that the indicator metric I can reflect the feasibility for magnetic navigation of a magnetic map. An area with larger I has a better positioning performance.

In the magnetic matching algorithm, the length L of the selected magnetic matching sequence is an important factor. L has a close relationship with the feasibility of magnetic navigation. In areas with high feasibility, a short magnetic matching length can lead to a good positioning result. In areas with low feasibility, L should be very large. Therefore, we attempt giving a suggested matching length L by using the comprehensive indicator metric I defined above. With an appropriate matching length, we can greatly improve the efficiency of the actual positioning process. For a magnetic map with a large amount of data, the matching length can be adjusted in a stepwise manner. In this way, the matching speed can also be effectively improved. Considering that the selection of the matching length L is also related to the performance and accuracy of the magnetometer, simulation experiments were carried out for analysis.

**Figure 9:**
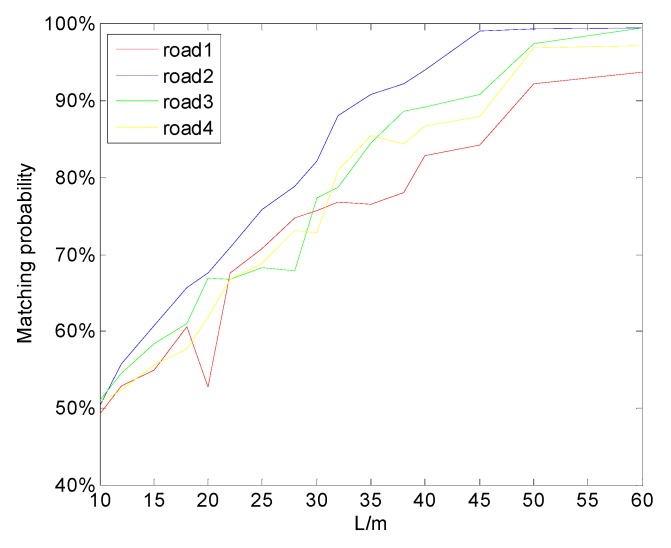
Matching probability under different matching lengths in the four road scenarios.

In order to investigate the impact of matching length acting on matching probability, Gaussian noise with variance of 3 μT was added to the above four magnetic maps. Matching experiments with different matching lengths were performed. The relationship between matching length and matching probability is shown in Figure 9. It can be seen that the matching probability was affected strongly by the matching length. It was almost linearly related to L when L was short, but it became saturated when L increased to a certain extent. The road segment with a larger I had a larger upper limit of matching probability, and the limit could be reached when L was shorter. When L was very short (L = 10 m), the matching probability of different road segments was about 50%, and the influence of road segment characteristics on matching probability was not obvious.

It can be seen from the test results that when I was 0.8996, 2.2392, 1.7519, and 1.0989, the corresponding L to achieve a matching probability beyond 85% was about 40, 30, 32, and 36 m, respectively. The L approximation was linear with 1/I; thus, a simple model was constructed as follows:

(22) $$L=\frac{a}{I}+b$$

**Figure 10:**
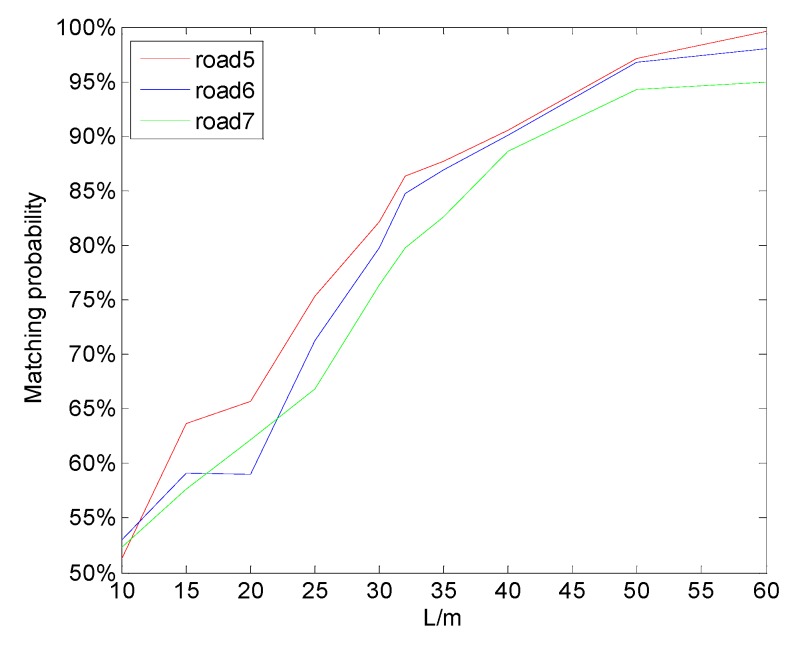
Matching probability under different matching lengths in three other road scenarios.

By fitting the first three segments of data above, we could get a=14.42,b=23.54. It was verified that the matching length of the fourth segment of road also satisfied the expression above. Therefore, L can be obtained using the above formula when the segment of road has a total length of 500 m and the matching probability is over 85%. For a magnetic map with a large amount of data, the map can be divided into some sub-graphs, and the matching length can be obtained on the sub-graphs. In order to verify the above conclusions, the other three magnetic maps were collected on the Beijing Fifth Circular Highway, and the corresponding magnetic characteristics were I = 1.9987, 1.5152, and 0.9832. The matching lengths recommended for the above road segments were L = 31, 34, and 39 m. The matching result with the noise of 3 μT is shown in Figure 10. It can be seen that, under the matching length proposed above, the three segments of road could obtain good matching positioning results. The matching length critical point with a matching probability of 85% was basically consistent with the proposed matching length. However, it was safer to take the matching length to 50 m or more without considering the matching time.

## 4. Special Influencing Factors in Magnetic Matching Navigation

In vehicle navigation, the measured magnetic field can be affected by special environmental factors. In order to explore the effects of these special influencing factors, the magnetic field strength in some special environments was measured and analyzed. Based on the comprehensive indicator metric I, we discuss the feasibility for navigation in these environments.

### 4.1. Magnetic Field Distribution around a Bridge

**Figure 11:**
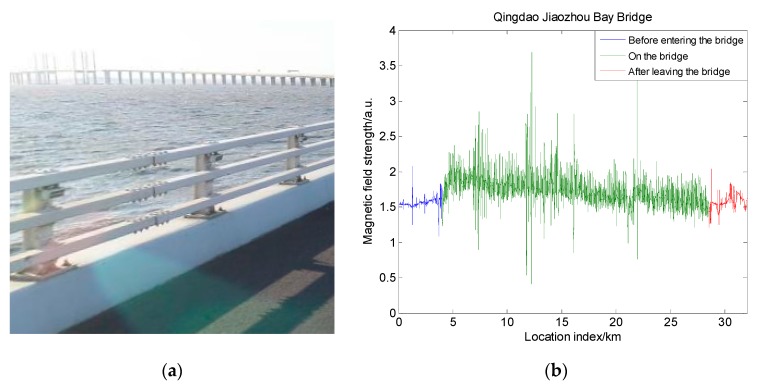
Field changes around Jiaozhou Bay Bridge: (**a**) schematic diagram of Jiaozhou Bay Bridge; (**b**) changes in magnetic field strength when passing the bridge.

The first analysis involved the impact of the bridge acting on the measured magnetic field. Since most modern bridge constructing materials include concrete and steel frames, they are magnetized by the earth’s magnetic field after construction. This is superimposed on the measured magnetic field. In order to make an intuitive analysis of the magnetic field distribution around the bridge, we obtained the magnetic field of the Jiaozhou Bay Bridge and its nearby areas. The Jiaozhou Bay Bridge is a cross-sea suspension bridge in Qingdao with a length of about 26 km, as shown in Figure 11a. It can be seen from the figure that the bridge is composed of a large amount of steel material, and it is surrounded by the sea. This means that, when the vehicle is driving on the bridge, it is not affected by nonexistent buildings. The main effect on the magnetic field is caused by the bridge itself. In the measuring process of the magnetic field, the magnetic sensor device used was SBGIG-500N. The measurement results are shown in Figure 11b. 

**Figure 12:**
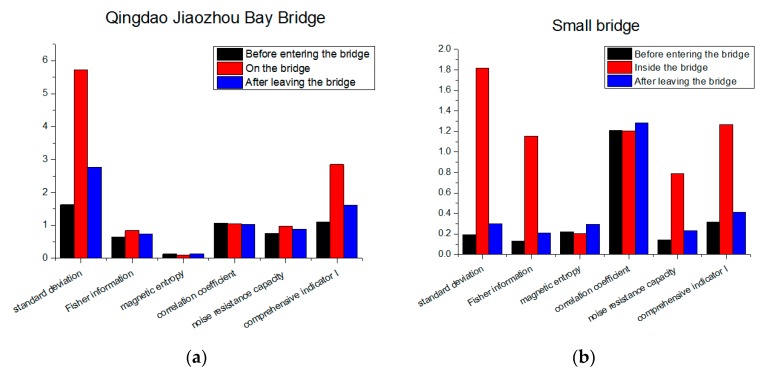
Characteristics and indicator I at different situations: (**a**) Qingdao Jiaozhou Bay Bridge; (**b**) a small bridge.

From Figure 11b, we can see that, before entering the bridge (blue line), the magnetic field distribution showed a stable trend. After entering the bridge (green line), the measured magnetic field strength began changing violently. It showed obvious characteristics at different positions, even at adjacent positions. When passing through the suspension cable area on the bridge, the measured magnetic field strength had a distinct peak, and the peak value could even reach three times the peak value of the magnetic field strength before entering the bridge. After leaving the bridge, the magnetic field strength returned to a smooth level from the earlier large fluctuation. To analyze the influence of the fluctuation changes on magnetic matching feasibility, we calculated the comprehensive indicator I in the three scenarios. We unified the magnetic measurements in order to compare them. The result is shown in Figure 12a. It can be found that, in the areas with obvious magnetic characteristics, the standard deviation of the magnetic map increased significantly. Other characteristics like the Fisher information and correlation coefficient did not have an obvious change. As a result, the comprehensive indicator I had an obvious increase on the bridge, and it returned to normal value after leaving the bridge. It can also be concluded that the bridge had a significant and continuous influence on the magnetic field strength. In vehicle navigation, the measured magnetic field will have obvious mutations when the vehicle passes the bridge. Thus, the bridge can be used as a characteristic mark in magnetic matching navigation to help determine the position of the vehicle. Moreover, it increases the feasibility for magnetic navigation.

**Figure 13:**
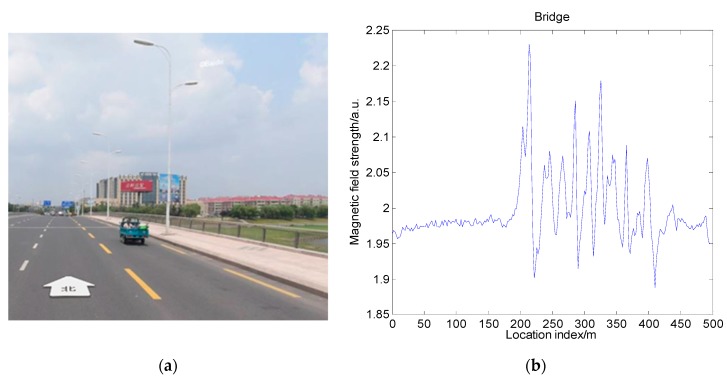
Field changes around a small bridge: (**a**) schematic diagram of the bridge; (**b**) change un magnetic field strength when passing the bridge.

The above analysis was aimed at large bridges such as Jiaozhou Bay Bridge; thus, the magnetic data were also collected and analyzed for small bridges. Figure 13a shows a certain short bridge in Beijing with a length of about 200 m. The change in magnetic field strength when a vehicle passed through the bridge is shown in Figure 13b. Due to the size of the bridge, the magnetic field changes caused by the bridge were smaller but more intuitive than those of the Jiaozhou Bay Bridge. We can see that the magnetic field was stable in the open area before entering the bridge, but it became irregular and fluctuated after entering the bridge. When the vehicle left the bridge, the magnetic field strength returned to the smooth value before entering the bridge. This shows that even small bridges can cause dramatic changes in the magnetic field. Furthermore, this change is a kind of mutation, instead of a gradient. This means that the influence of the bridge decreased rapidly with the distance, and its range was small. This further verifies the feasibility of using the bridge as a characteristic sign. When strong fluctuation of the magnetic field strength is detected during vehicle navigation, it can be judged whether there is a bridge around the rough position of the vehicle. This improves the positioning accuracy of the vehicle. To discuss the effects on feasibility for magnetic navigation, we calculated the comprehensive indicator metric I. The result is shown in Figure 12b. From the figure, we can see that the changes in magnetic field strength can be well described by the changes in comprehensive indicator metric I. We can conclude that, just like Jiaozhou Bay Bridge, even small bridges have a significant influence on the feasibility for magnetic navigation.

### 4.2. Magnetic Field Distribution around a Tunnel

**Figure 14:**
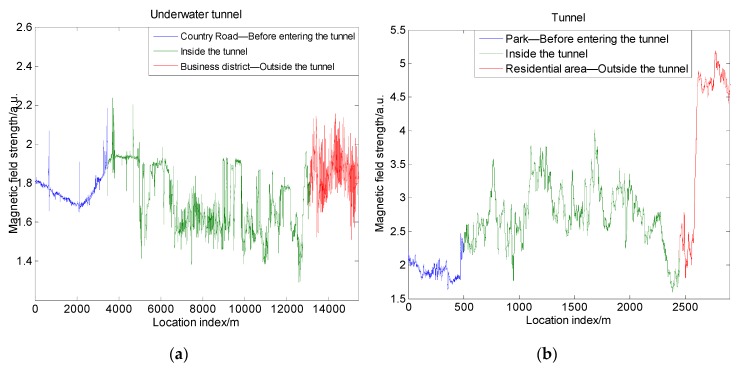
Field changes when passing through a tunnel: (**a**) Jiaozhou Bay Underwater Tunnel; (**b**) Datun Road Underground Tunnel.

A tunnel is also a special static environment when driving a vehicle. In a tunnel, GNSS navigation loses its dominance. At this time, the magnetic navigation method can play an important role in the tunnel. Therefore, it was necessary to analyze the magnetic field distribution around the tunnel. We collected the magnetic field strength of the Jiaozhou Bay Underwater Tunnel in Qingdao and the Datun Road Tunnel in Beijing. Jiaozhou Bay Underwater Tunnel is a submarine tunnel with a total length of about 7.8 km. When the vehicle is under the sea, it can be regarded that the measured magnetic field is only affected by the tunnel itself. Outside the tunnel exit is a business district, and the measured magnetic field is affected by a large number of buildings. The Datun Road Tunnel is an underground tunnel located near the Beijing Olympic Park, with a length of about 2 km. Outside the tunnel exit is a residential area, and the measured magnetic field may also be affected by buildings. Figure 14a,b shows the measured magnetic field strength diagrams when the vehicle passed through the two tunnels. 

Similarly to the bridge case, the magnetic strength value before entering the tunnel was stable and had dramatic changes inside the tunnel. Compared to the underwater tunnels, underground tunnels have more severe fluctuation, which may be related to the magnetic material under the ground. Moreover, the steady and irregular fluctuations of the magnetic strength changes occurred successively in the underwater tunnel, unlike the continuous fluctuation of the underground tunnel. In addition, the trend of the changes in magnetic strength in the tunnel had a spatial gradient characteristic. After leaving the tunnel, the magnetic field strength firstly returned to the steady value before entering the tunnel. Then, the vehicle entered the area where buildings are distributed densely, such as commercial areas and residential areas, and then the magnetic field strength had a significant change. 

**Figure 15:**
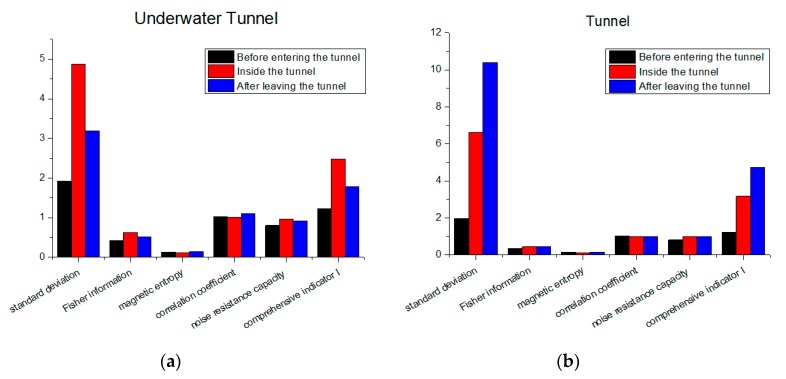
Characteristics and indicator I in different situations: (**a**) Jiaozhou Bay Underwater Tunnel; (**b**) Datun Road Underground Tunnel.

To explore the changes in magnetic matching feasibility, we calculated the magnetic characteristics and comprehensive indicator I. The result is shown in Figure 15. We can see that the effects of tunnels were more obvious in terms of standard deviation. As a result, the indicator metric I also had a significant increase. This shows that the matching feasibility increased due to the influence of the tunnel. At the exit of the tunnel, we can see that the influence caused by the buildings acting on the magnetic field strength was particularly significant. Because the building distribution at the exit of the short tunnel (Figure 15b) was denser than that at the exit of the long tunnel (Figure 15a), the indicator metric I at the exit of the short tunnel was bigger. When the vehicle was inside the tunnel and at the exit of the tunnel, the indicator I was much bigger than that before entering the tunnel. This result shows that the tunnel has a similar impact on magnetic matching feasibility to buildings. The magnetic maps with tunnels have bigger matching feasibility. Moreover, in the magnetic navigation of the vehicle, whether the vehicle passes through the tunnels can be inferred from whether the magnetic field strength change is abrupt. This can benefit magnetic matching navigation.

### 4.3. Magnetic Field Distribution in an Underground Garage

**Figure 16:**
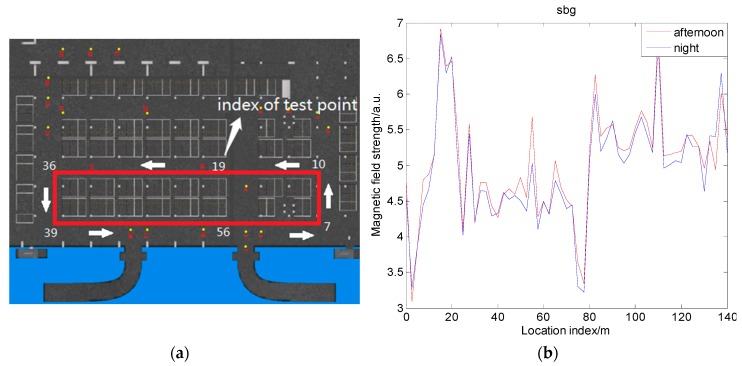
Field distribution in an underground garage: (**a**) test scene and route in garage; (**b**) changes in magnetic field strength in the garage.

A very important application scenario for vehicle navigation is an underground garage. An underground garage is a confined space, where the application of GNSS has limitations. Thus, we considered using magnetic navigation to overcome this deficiency. However, in underground garages, the parking situation of vehicles is unstable. If a parked vehicle has a significant influence on the magnetic field, the same location of the underground garage in different parking situations will have different magnetic field strength values, resulting in a limitation of the magnetic matching navigation. In order to explore the differences of the magnetic field in different vehicle parking situations, an experiment was designed. A series of test points were established in the underground garage area of a research institute as shown in Figure 16a. The distance between the test points was 2.5 m. To carry out the magnetic field measurement in the actual positioning process, the test points were built on the center line of the garage walkway. For the purpose of obtaining an accurate magnetic field strength value, about 2000 values were measured at each test point, and the average value was taken as the final value. The two parking situations involved when vehicles were parked intensively in the afternoon, and when there were almost no vehicles parked in the evening. The result of the experiment is shown in Figure 16b. The experimental equipment used was SBGIG-500N. 

As can be seen from the figure, the measured magnetic envelopes of the underground garage in the different vehicle parking situations were almost the same. Moreover, the two measured magnetic field strength values were partially different when the index of the test point was 19–36 in Figure 16a (location index of 47–90 m in Figure 16b). This phenomenon may be possible due to the space area being relatively closed and the vehicle parking being crowded at 19–36; thus, the magnetic field was simultaneously distorted by the vehicles on both sides. However, the difference was relatively small compared to the magnetic value of the test point itself. The positioning tests where data were collected twice show that the difference caused by different parking environments had little effect on the positioning result after selecting an appropriate matching length; thus, it can be inferred that the magnetic matching positioning method is feasible even in different vehicle parking environments, and it can be used in a garage.

### 4.4. Impact of Electronic Devices in the Vehicle

**Figure 17:**
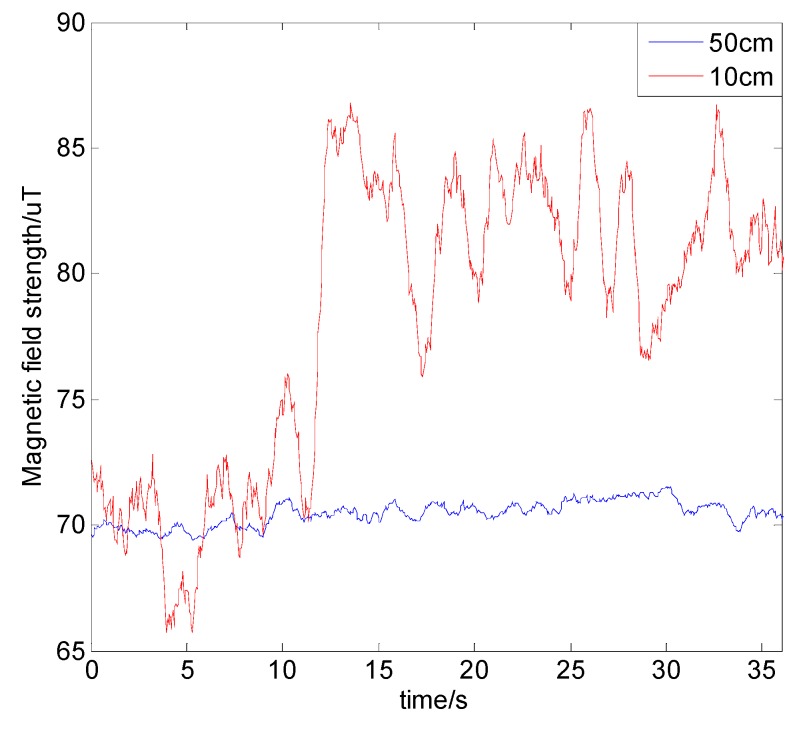
Magnetic field strength when a nearby chassis is turned on.

Since the magnetic sensor is installed in the vehicle, the measured magnetic field is also affected by the electronic equipment in the vehicle. The steel material of electronic devices can be magnetized by the earth’s magnetic field, and, if the electronic device is powered on, the current will generate an induced magnetic field around it. In order to analyze the magnitude and range of the induced magnetic field, we measured the magnetic field strength at the positions of 50 cm and 10 cm from a chassis on the vehicle, and then powered on the chassis. The results are shown in Figure 17, and the power-on test of the chassis was performed at around 10 s. It can be seen that the magnetic field strength at 50 cm from the chassis was barely affected by the chassis, and the power supply of the chassis also had little effect. At 10 cm from the chassis, the magnetic field value of the testing point fluctuated. After the power turned on, the magnetic field strength increased greatly and changed continuously, and a stable state could not be reached. The amplitude of fluctuation also increased significantly compared to the fluctuation before power-on. Therefore, it can be concluded that the electronic devices in the vehicle have a continuous and significant influence on the magnetic field, but this decays rapidly with distance, and it is out of the influence range beyond 50 cm. Thus, in the actual vehicle navigation process, the magnetic sensors should be placed away (at least 50 cm) from electronic devices and steel materials to eliminate their influence on the magnetic field.

### 4.5. Impact of Sensor Installation 

During the measuring process of the magnetic field, the installation state of the sensors cannot be guaranteed to be exactly the same. Therefore, it is necessary to analyze whether the sensor installation state can affect the measured magnetic field.

#### 4.5.1. Impact of Installation Angle

**Figure 18:**
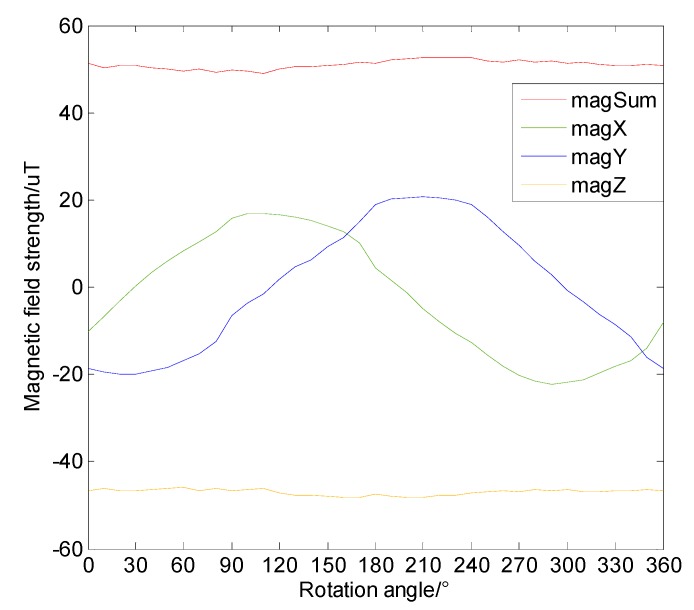
Magnetic field strength when the sensor’s azimuth angle changes.

The first characteristic we analyzed was the effect of the installation angle of the sensor acting on the measured magnetic field. The installation angle of the sensor is mainly divided into an azimuth angle, a pitch angle, and a roll angle. In the actual vehicle navigation process, the magnetic sensor is generally fixed on a platform in the vehicle; thus, the only installation angle we considered was the azimuth angle. In order to investigate the difference of the measured magnetic field in the case of azimuth direction angles, an experiment was designed. The magnetic sensor was fixed at a center point and rotated 360° around the center point. Each of the test points rotated by 10°, providing 36 points in total. About 1000 magnetic field values were measured per point, and the averaged value was regarded as the true magnetic value of the point. The measurement results are shown in the Figure 18, where the horizontal axis shows the rotation angle, which is the azimuth angle, while the vertical axis shows the measured magnetic field strength. It can be seen that, as the azimuth angle increased, the magnetic field in both the *x*-axis and the *y*-axis had a large change, and, due to the orthogonality of the *x*-axis and *y*-axis, the magnetic fields also had symmetry. Since the rotation occurred on the *xy* plane, the *z*-axis magnetic field did not change substantially, and the combined magnetic field strength was also stable. Therefore, to ensure the consistency of magnetic map and test data in the magnetic matching process, and to avoid the influence of the sensor installation angle, the combined magnetic field strength value should be taken as the magnetic field value of a certain position rather than the three-axis magnetic field value.

#### 4.5.2. Impact of Installation Height

**Figure 19:**
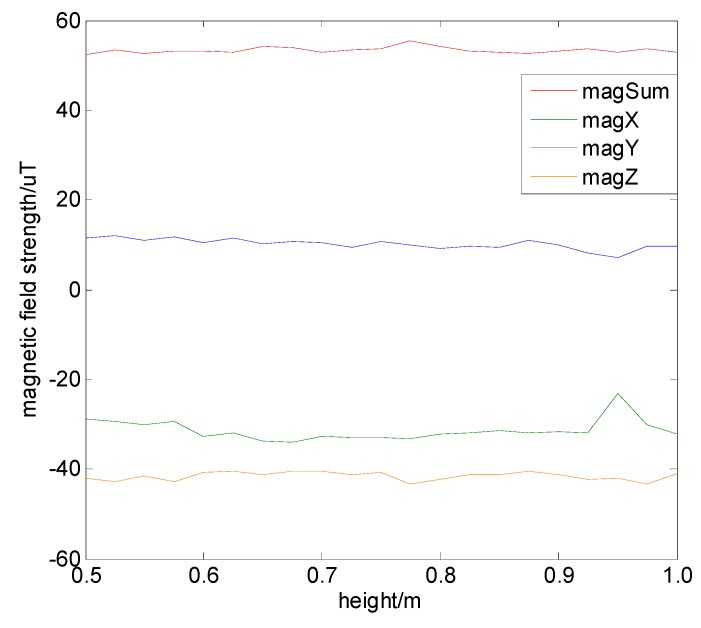
Magnetic field strength when the sensor’s installation height changes.

When positioning experiments are performed on different vehicles, the installation height of the magnetic sensor may also have differences due to the size of the vehicle. Thus, the next step was to investigate the impact of the installation height acting on the measured magnetic field. We designed an experiment to test this. On the same vertical line of 1 m, the test points were set every 5 cm, and 1000 magnetic values were measured at each point. We chose the average magnetic value as the final value. It should be noted that the sensor installation angle should be kept constant during the measuring process, and the measurement results are shown in Figure 19. It can be seen that during the process of changing the installation height, the three-axis magnetic field strength and the combined magnetic field strength did not change significantly. We can infer that the magnetic field strengths at the same location measured on different vehicles are consistent, which increases the practicality of magnetic positioning in vehicle-oriented applications.

#### 4.5.3. Impact of Installation Platform

**Figure 20:**
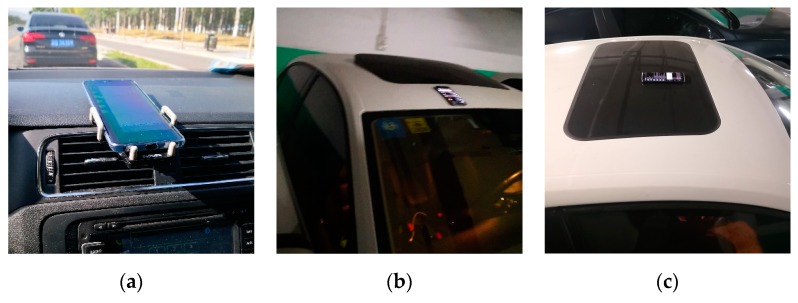
Installation platforms: (**a**) suspended installation; (**b**) installation on iron shell; (**c**) installation on glass shell.

**Figure 21:**
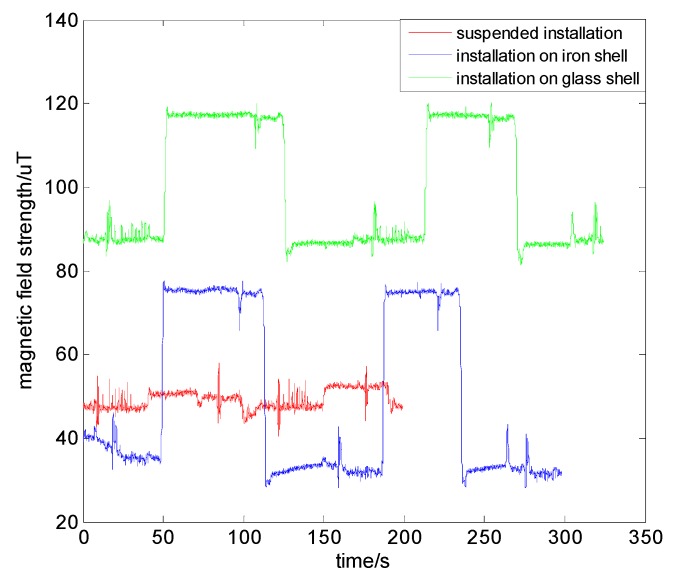
Magnetic field on different installation platforms.

In the actual positioning process of magnetic navigation for vehicles, the installation platform of magnetic sensors may affect the magnetic measurement results. Therefore, we designed experiments to explore the effects of different installation platforms on the measurement of the magnetic field. We discussed three kinds of situations: when the magnetic sensors are suspended, when they are on an iron shell, and when they are on a glass shell, as shown in Figure 20. The vehicle traveled two laps along the same road, and the magnetic data obtained are shown in Figure 21. It can be seen that the magnetic measurement results of different installation platforms were obviously different. This may be due to the ferromagnetic shell of the vehicle. The two laps of magnetic data collected on the same platform were consistent. Moreover, the magnetic characteristics of the suspended magnetic sensor were less obvious than those of the iron shell. We can infer that the magnetic field generated by the ferromagnetic shell is small in the suspended magnetic sensor position, and it is almost attenuated. Also, since the vehicle shape used in the magnetic map constructing phase and in the positioning phase are likely to be different, we should minimize the influence of the ferromagnetic shell on the measurement to ensure the consistency of the data in the two phases. Therefore, when installing the magnetic sensor, we hope to be able to have a suspended installation in the center of the vehicle.

### 4.6. Impact of Sensor Accuracy 

The accuracy of the sensor is also an important impact factor when measuring magnetic fields. Sensors with lower accuracy may not be able to detect changes in the magnetic field with characteristic information, which is not conducive to positioning. However, a sensor with higher precision is more likely to detect the interference of the surrounding environments, which brings additional errors to the positioning results [29]. At the same time, there may be a gap between the magnetic maps collected by different sensors in the same region. This can also affect the magnetic positioning results. 

**Figure 22:**
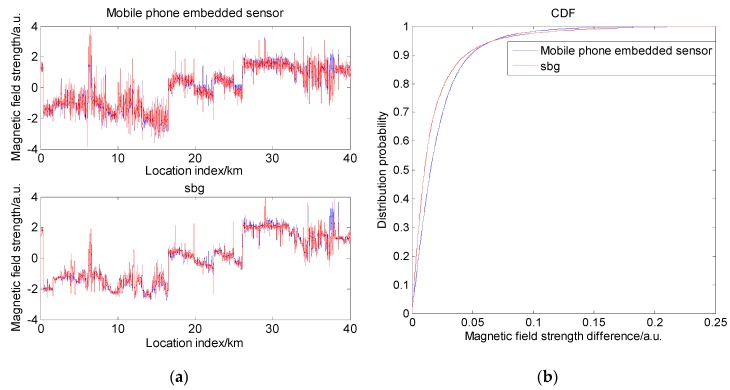
Same magnetic map acquired twice by two different sensors: (**a**) magnetic field strength comparisons of two sensors; (**b**) similarity of the magnetic maps collected twice.

In order to explore the effects of different sensor accuracy, the magnetic field strengths were collected using the Huawei Mate8 mobile phone-embedded sensor and the SBGIG-500N sensor in the same road scenarios around Beijing. It should be noted that different types of magnetic sensors have different measurement biases; thus, the magnetic sensors should be calibrated before collection [30]. The normalized result is shown in Figure 22a, where the blue and red lines in the figure are the results of two magnetic maps of the same road segment at different times. It can be seen that the envelopes collected by the two sensors had similarities, but there were also gaps in some scenarios. From Figure 22a, it can be seen that the SBG sensor had weaker magnetic fluctuation than the embedded sensor of the mobile phone, indicating that SBG has stronger anti-noise ability. Figure 22b shows the similarity of the blue line and red line. The horizontal axis shows the magnetic strength difference of the corresponding points of the two magnetic lines, and the vertical axis shows the probability of the magnetic strength being in a specific range. It can be seen that the probability within the same range of magnetic strength of SBG was larger, indicating that SBG had a greater similarity between magnetic field values acquired twice compared to the embedded sensor of the mobile phone. This means that SBG has better stability, which is beneficial for positioning. To estimate the matching feasibility of the two magnetic maps, the comprehensive indicator I was calculated. The result is shown in Table 3. From the table, we can see that the two magnetic maps had similar characteristics, but SBG had a larger standard deviation and better anti-noise ability. This would result in magnetic maps of SBG having better magnetic matching feasibility than a mobile phone and, thus, better performance in actual magnetic positioning.

### 4.7. Spatial Resolution and Time Stability 

#### 4.7.1. Spatial Resolution

**Figure 23:**
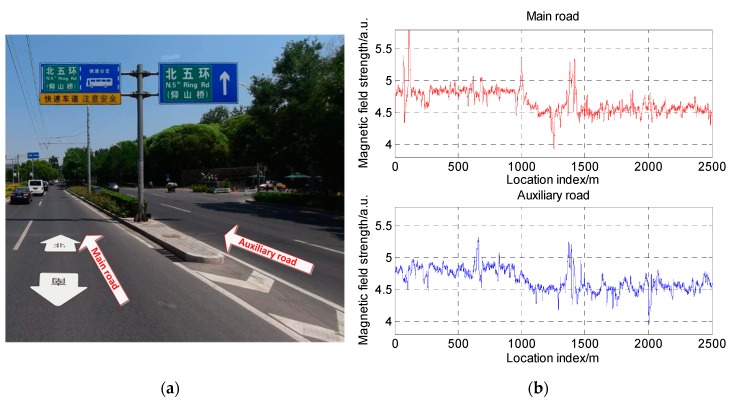
Field distribution on a main road and auxiliary road: (**a**) schematic diagram of the two roads; (**b**) comparison of the magnetic maps of the two roads.

From Section 3.4, we can see that roads in different scenarios have totally different magnetic fields. This spatial resolution can be used to distinguish different roads. However, there is a lack of research on the scale of spatial resolution. If adjacent roads have different characteristics, we can locate the vehicle exactly in the lane. To carry out a more detailed analysis of the spatial resolution of the magnetic field, we designed magnetic field acquisition test on a main road and an auxiliary road as shown in Figure 23a. The magnetic field measurement results are shown in Figure 23b.

As can be seen, the data of the two roads had a certain similarity as a whole. However, in terms of details, the position and value of the magnetic field peaks were slightly different, which means that every different road has unique characteristics. In order to distinguish the magnetic data of two roads, the magnetic segments selected for distinguishing should be long enough, in order to make sure that the two magnetic segments have a large difference. Therefore, it is possible to consider this difference to distinguish the road the vehicle is on to improve vehicle positioning accuracy.

#### 4.7.2. Time Stability

**Figure 24:**
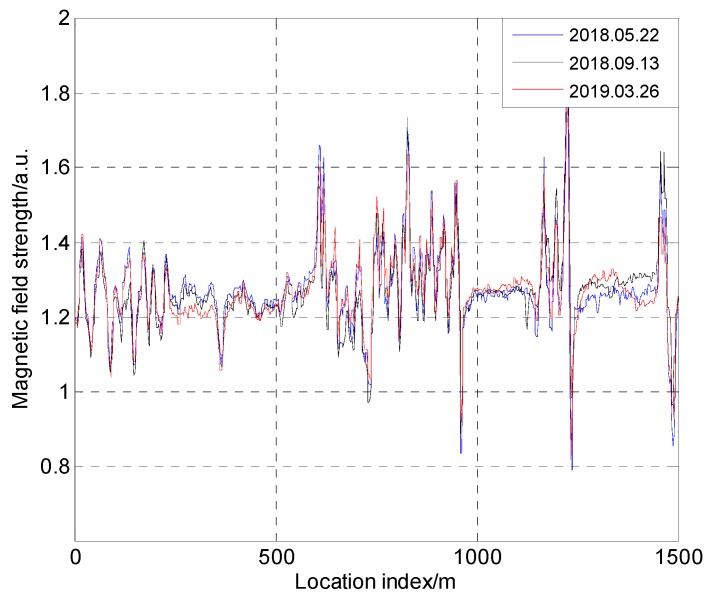
Magnetic field map collected at different times.

Since the magnetic strength of a location area is closely related to static environments such as building distributions, if there are large terrain changes (such as building construction) in the area, an updated magnetic map will be required. In order to investigate whether the magnetic map changes if large terrain changes do not exist, we designed an experiment. Magnetic data were acquired over a half-year span on the G7 highway in Beijing, using the SBGIG-500N sensor. The experimental results are shown in Figure 24. It can be seen that the magnetic field strength had strong similarity at different time nodes. This time stability of the magnetic field increases the feasibility of magnetism for positioning. Based on the research on buildings and bridges, we can see that, when there are no large geomorphological changes, it is not necessary to update the magnetic map. When changes occur, whether the magnetic map should be updated can be judged based on the distance of the source from the changes and the road. 

## 5. Conclusions and Future Work

In this paper, we proposed a model to discuss the magnetic field around environmental objects such as buildings, bridges, and vehicles. The model focused on the relationship between the value of the magnetic field generated by the object and the distance away from the object. The experimental results show that the actual magnetic distribution is consistent with the model, which confirms the availability of the model. This model can also be used to easily analyze the feasibility of magnetic navigation. Furthermore, we presented a comprehensive indicator metric to quantitatively evaluate the feasibility of magnetic navigation. The metric is based on five important statistical characteristics of magnetic maps. According to the metric, we proposed a method for evaluating the appropriate magnetic matching length. Experiments using scenes with different building densities prove that the metric can give an appropriate description of the feasibility of magnetic navigation. Also, good positioning results can be obtained under the matching length we recommended. At last, we discussed the magnetic impact on the feasibility of magnetic navigation caused by ground factors, which was intuitively reflected in the metric we presented. In this way, we can estimate the magnitude and scope of the impact and compensate for the impact in a targeted way. This can be used to improve positioning accuracy.

Future work can focus on adjusting this comprehensive indicator and improving the estimating model of matching length. We may use a more complicated model to give the recommended matching length. Moreover, deciding which lane the vehicle is in is also a major challenge. For areas with sparse sources, we can work on detecting and compensating for the interference impacts. There are methods of establishing a mathematical model to compensate for the interference impact [31], but the lack of comprehensive analysis of the interference source is also a problem. Follow-up research could be carried out to solve this problem.

## Figures and Tables

**Table 1:** Importance matrix construction standard.

Comparation of Factor *i* to Factor *j*	Equally Important	Slightly Important	Medium Strongly Important	Strongly Important	Extremely Important	Intermediate Value of Two Adjacent Judgments
Quantitative value	1	3	5	7	9	2, 4, 6, 8

**Table 2:** Matching probability after adding Gaussian white noise to the four roads.

Testing Roads	Gaussian White Noise (0, 1)	Gaussian White Noise (0, 3)	Gaussian White Noise (0, 5)	Gaussian White Noise (0, 10)
Scenario 1 (I = 0.8996)	88.24%	64.41%	39.02%	6.14%
Scenario 2 (I = 2.2392)	99.69%	93.34%	67.95%	25.81%
Scenario 3 (I = 1.7519)	94.59%	75.55%	53.8%	16.02%
Scenario 4 (I = 1.0989)	89.49%	66.1%	46.51%	9.05%

**Table 3:** Comprehensive indicators of the two magnetic maps.

	Standard Deviation	Fisher Information	Magnetic Entropy	Correlation Coefficient	Anti-Noise Ability	I
Mobile phone	12.6059	0.6303	0.0883	1.0044	0.9838	5.6291
SBG	13.7762	0.3466	0.0884	1.0003	0.9955	6.0829
